# Electroclinical Dissociation in Generalized Epilepsy: A Video‐EEG Case From Sub‐Saharan Africa

**DOI:** 10.1155/carm/5593355

**Published:** 2026-08-12

**Authors:** Yihealem Yabebal Ayele

**Affiliations:** ^1^ Department of Internal Medicine, College of Medicine and Health Sciences, Bahir Dar University, Bahir Dar, Ethiopia, bdu.edu.et

**Keywords:** child and adolescent psychiatry, electroclinical dissociation, functional dissociative seizures, functional neurological disorder, genetic generalized epilepsy, video-electroencephalography

## Abstract

**Introduction:**

Functional neurological symptoms, specifically functional dissociative seizures (FDS), frequently coexist with epilepsy and pose severe diagnostic challenges, particularly in resource‐limited settings where video‐electroencephalography (video‐EEG) access is scarce. Overreliance on interictal or noncausal epileptiform EEG abnormalities without rigorous electroclinical correlation risks misdiagnosis and hazardous escalation of antiseizure medications (ASMs).

**Case Presentation:**

We report a 17‐year‐old male from rural Ethiopia with a 4‐year history of genetic generalized epilepsy (GGE) and mild intellectual impairment who presented with persistent, variable left lower limb jerking following a road traffic accident involving the same limb. Video‐EEG captured the jerking episodes and demonstrated generalized spike–wave and polyspike–wave discharges (3–4 Hz) that coincided temporally with clinical jerks but lacked electrographic evolution, focal cortical origin, or muscle phase‐locking on surface recording. The events occurred exclusively during wakefulness with preserved awareness, no autonomic instability, and no postictal state. Sequential ASM trials (levetiracetam, valproate, clonazepam, and phenobarbital) failed to modulate the jerks. The electroclinical presentation was interpreted as electroclinical dissociation, confirming coexisting generalized epilepsy and FDS. The patient had also undergone posterior fossa decompression for Chiari I malformation 9 days prior, which was excluded as a cause for the motor events based on clinical and radiological stability.

**Conclusion:**

Diagnostic evaluation of FDS should rely on a positive “rule‐in” framework as recommended by international guidelines. Even when epileptiform discharges appear on EEG during clinical events, a lack of physiological congruence between electrographic patterns and semiology confirms electroclinical dissociation.

**Key Clinical Message**
• Temporal EEG abnormalities do not necessarily indicate epileptic causality. In patients with established epilepsy, persistent focal or unilateral motor events with preserved awareness and poor response to antiseizure medications should raise high suspicion for coexisting functional dissociative seizures (FDS). Careful video‐EEG electroclinical correlation is essential to prevent diagnostic overreliance on interictal patterns and avoid hazardous polypharmacy.

## 1. Introduction

Functional dissociative seizures (FDS), historically referred to as psychogenic nonepileptic seizures (PNES), are paroxysmal events characterized by altered motor, sensory, or cognitive function that resemble epileptic seizures but arise from altered brain network connectivity rather than ictal epileptiform discharges [1, 2]. In accordance with the recent consensus of the International League Against Epilepsy (ILAE) Task Force, the term FDS is preferred to reduce stigma, improve diagnostic communication, and establish nosological clarity [1].

FDS is increasingly recognized in pediatric and adolescent populations, frequently presenting with complex motor semiologies and somatic comorbidities [3, 4]. Contemporary epidemiological data indicate that 10%–30% of individuals diagnosed with FDS have coexisting epilepsy [2, 5]. This dual diagnosis is particularly difficult to navigate in low‐ and middle‐income countries (LMICs), where access to continuous video‐electroencephalography (video‐EEG) monitoring is constrained, and interictal epileptiform discharges are frequently misattributed as the sole generator of all paroxysmal events [5, 6].

Recent guidelines from the ILAE Pediatric Psychiatric Issues Task Force emphasize that FDS diagnosis should no longer be viewed as an exclusionary “diagnosis of last resort.” Instead, clinicians should utilize a positive “rule‐in” approach based on specific semiological features (e.g., preserved awareness during bilateral motor activity, variable movement frequency/amplitude, distractibility, and lack of postictal confusion) alongside video‐EEG demonstration of electroclinical dissociation [7, 8]. Furthermore, effective care demands a multidisciplinary approach bridging pediatric neurology, psychiatry, and physical rehabilitation [4, 8].

Physical trauma, chronic medical illness, structural central nervous system anomalies, and psychosocial stressors (such as legal litigation) serve as well‐established predisposing and precipitating factors for functional neurological disorders (FNDs) [3, 9]. Here, we present a complex case from sub‐Saharan Africa demonstrating electroclinical dissociation in a patient with genetic generalized epilepsy (GGE) who developed FDS localized to an injured limb following trauma and posterior fossa surgery.

## 2. Case History

### 2.1. Personal, Developmental, and Socioeconomic Background

The patient is a 17‐year‐old male residing in a rural agricultural community in southern Ethiopia. He was born via an uncomplicated full‐term vaginal delivery at home without documented perinatal asphyxia or neonatal intensive care admission. Early psychomotor developmental milestones (gross motor, fine motor, and language) were reported by his parents as normal. However, upon entering formal schooling, he exhibited significant learning difficulties, repeating multiple grades, and achieving only a Grade 5 educational level by Age 17.

Formal psychometric evaluation (e.g., WAIS‐IV) could not be performed due to the lack of validated, culturally adapted testing tools in the regional setting. However, based on clinical history, adaptive functioning, and educational trajectory, a clinical diagnosis of borderline intellectual functioning to mild intellectual disability was established. Regarding autonomy, the patient is fully independent in basic activities of daily living (ADLs) including dressing, eating, and personal hygiene but requires family supervision for complex instrumental ADLs (such as managing finances or navigating unfamiliar travel).

Physiological habits revealed regular sleep–wake schedules (7–8 h/night) without parasomnias, though mild undernutrition (BMI 17.2 kg/m^2^) was noted. Medical history was negative for prior psychiatric hospitalization or childhood abuse. Family history was noncontributory for epilepsy, febrile seizures, or psychiatric disorders among first‐ or second‐degree relatives. The family belongs to a low socioeconomic stratum, and significant financial strain was present, compounded by ongoing legal litigation seeking financial compensation following a motor vehicle accident.

### 2.2. Characterization of Epilepsy

The patient’s epilepsy began at Age 13 (4‐year total duration). His primary seizure semiology consisted of episodic generalized tonic‐clonic seizures (GTCS) characterized by a sudden loss of consciousness, eye revulsion, tonic stiffening followed by clonic jerking of all four extremities, tongue biting, and urinary incontinence, lasting 1–3 min. Seizures were followed by a 30–60‐min postictal state of confusion and deep somnolence. Prior to the onset of functional events, his GTCS frequency was approximately 1–2 events per month.

According to the ILAE 2017 Classification of the Epilepsies, his diagnosis was classified as GGE/idiopathic generalized epilepsy (IGE) with GTCS alone. The initial diagnosis was made at Age 14 by a consultant pediatrician at a regional referral hospital based on classic clinical semiology and an interictal EEG demonstrating generalized 3‐Hz spike‐and‐wave discharges. Historical neuroimaging (brain CT at Age 14) was unrevealing.

### 2.3. Index Presentation, Physical Trauma, and Surgical Context

Three months prior to his current evaluation, the patient was involved in a road traffic accident where a light vehicle ran over his left lower leg. He sustained soft‐tissue contusions and a nondisplaced fibular crack fracture, which was managed conservatively with transient immobilization. Shortly after the injury, he developed persistent, involuntary jerking movements confined exclusively to the left lower limb. The jerks occurred multiple times daily during wakefulness.

Concurrently, the patient developed prominent somatic symptoms, including episodic dysphagia to solids, chronic daily tension‐type headaches, and diffuse neck pain. Psychological screening utilizing clinically adapted PHQ‐9 and GAD‐7 questionnaires revealed moderate depressive symptoms (PHQ‐9 score: 14) and prominent health‐related anxiety (GAD‐7 score: 13), driven by somatic amplification and distress surrounding the slow recovery of his leg and the active legal court case against the driver. These somatic complaints were interpreted as potential clinical correlates and “red flags” reflecting underlying somatic symptom burden and affective distress, which frequently act as vulnerability factors precipitating FND.

Workup for his chronic neck pain and headache led to brain and cervical magnetic resonance imaging (MRI), which revealed a classic Chiari I malformation with 8‐mm cerebellar tonsillar herniation below the foramen magnum without syrinx. Nine days prior to his transfer to our neurology unit, he underwent uneventful posterior fossa decompression surgery. His postoperative recovery was smooth, with resolution of neck pain and no operative complications.

### 2.4. Clinical Examination

Upon transfer to our unit, physical examination revealed a conscious, oriented young male (Glasgow Coma Scale 15/15). Neurological examination between motor events was unremarkable: cranial nerves were intact, muscle strength was 5/5 in all four extremities, deep tendon reflexes were 2+ bilaterally, and plantar responses were flexor. There were no signs of myelopathy, cerebellar ataxia, or meningeal irritation. Examination of the left lower extremity revealed no focal muscle atrophy, joint contracture, or localized motor deficit.

During clinical observation, the left lower limb jerking demonstrated marked semiological variability. The movements ranged from rapid, low‐amplitude myoclonic‐like jerks to rhythmic, high‐amplitude coarse flexor movements at the hip and knee. The events occurred exclusively while awake and were noticeably accentuated when attention was drawn to the limb but diminished during complex cognitive tasks (e.g., forward digit span). Consciousness remained completely preserved throughout all events, with no speech arrest, no facial involvement, no autonomic instability (tachycardia and diaphoresis), and no postevent confusion.

### 2.5. Differential Diagnosis

The differential diagnosis included focal motor status epilepticus/epilepsia partialis continua (EPC), epileptic myoclonus, spinal/peripheral myoclonus, postsurgical cervical cord irritation, and FDS.

Focal motor seizures/EPC: Excluded due to the absence of contralateral focal cortical discharges, lack of Jacksonian march, strict variability in movement frequency/amplitude, distractibility, and total lack of response to high‐dose antiseizure medications (ASMs).

Postsurgical complication/cervical cord irritation: Given the surgical decompression performed 9 days prior, spinal cord compression, CSF leak, or posterior fossa surgical irritation were considered. This was excluded because of the following: (a) the left lower limb jerks originated 3 months prior to surgery immediately following the local limb trauma; (b) postoperative cervical spine MRI demonstrated successful decompression without spinal edema, hemorrhage, or syrinx; and (c) the patient lacked hyperreflexia, clonus, or Hoffmann/Babinski signs.

Spinal/peripheral myoclonus: Excluded due to the absence of stimulus sensitivity (tactile or acoustic triggering), lack of fixed rhythmicity, persistence during active mental distraction, and normal peripheral nerve conduction studies.

FDS: Confirmed based on positive “rule‐in” semiological features (distractibility, high variability, limb localization to prior trauma site, and preserved awareness) coupled with video‐EEG proof of electroclinical dissociation.

### 2.6. Investigations

Continuous video‐EEG monitoring was conducted for 24 h, capturing multiple spontaneous clinical episodes of left lower limb jerking.

Basal (interictal) EEG findings: The background rhythm during quiet wakefulness demonstrated a symmetric, reactive 9‐Hz posterior dominant alpha rhythm. Intermittently during baseline sleep and wakefulness, brief (1–2 s) generalized, bilaterally synchronous spike‐and‐wave and polyspike‐and‐wave discharges (3–4 Hz) were noted without clinical motor manifestations.

**FIGURE 1:**
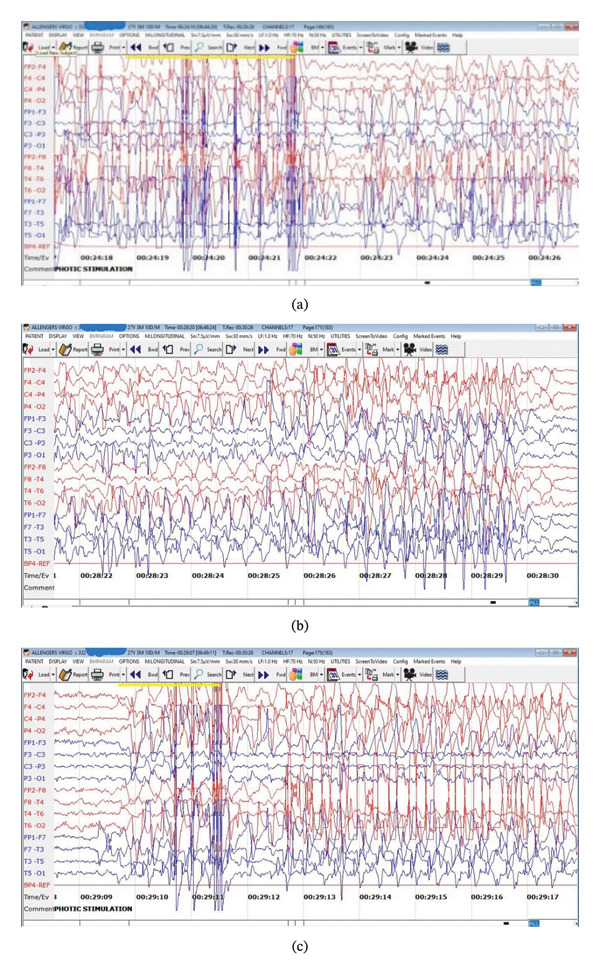
(a–c) EEG traces showing generalized spike–wave activity during focal limb movement without electrographic evolution.

Ictal/event video‐EEG analysis: During active left lower limb jerking, the EEG demonstrated bursts of generalized spike–wave discharges (GSWDs) (Figure 1a–c). To rigorously evaluate the relationship between these discharges and the motor jerks, surface electromyography (EMG) monitoring was applied over the left rectus femoris and anterior tibialis muscles. Visual inspection confirmed that there was no consistent, phase‐locked time relationship between the cortical GSWD spikes and the lower limb muscle bursts. The GSWD showed no focal cortical onset, no lateralization to the right frontoparietal cortex, no focal frequency attenuation, and no postevent cerebral slowing.

Interpretation: The presence of generalized discharges during focal motor events represents a noncausal co‐occurrence (electroclinical dissociation). The underlying GSWD reflects the patient’s baseline GGE traits, whereas the focal limb jerking is generated by nonepileptic, functional mechanisms.

Laboratory workup: Complete blood count, serum electrolytes, liver and renal panel, serum calcium, magnesium, thyroid function, and inflammatory markers (ANA and RF) were within normal limits.

### 2.7. Treatment and Clinical Course

Following video‐EEG confirmation of electroclinical dissociation, ASMs were rationalized. Unnecessary acute escalations (short‐term phenobarbital and clonazepam) were systematically tapered and discontinued. His underlying GGE was maintained on valproic acid (500 mg twice daily) and levetiracetam (750 mg twice daily).

### 2.8. Management of FDS and Comorbidities

Psychoeducation: A structured, transparent diagnostic explanation was delivered jointly by the neurology and psychiatric teams to the patient and his family. The diagnosis of FDS was explained positively (“the brain’s software is miscommunicating following trauma and stress, rather than permanent hardware damage”).

Indication for pharmacotherapy: Fluoxetine (20 mg daily) was initiated. It is critical to clarify that fluoxetine was prescribed explicitly for his co‐occurring major depressive episode and prominent health‐related anxiety (PHQ‐9: 14, GAD‐7: 13) triggered by physical trauma and legal stress, rather than as a primary direct therapy for FDS. Existing evidence indicates limited efficacy for SSRIs in functional movement disorders in the absence of comorbid mood or anxiety disorders [10].

Physical rehabilitation and psychotherapy: The patient commenced physical therapy focusing on motor retraining, unlearning abnormal movement patterns, and grounding techniques. Outpatient cognitive behavioral therapy (CBT) tailored for FDS was initiated.

Follow‐up outcomes: At the 2‐month outpatient follow‐up, his epileptic GTCS remained fully controlled. The frequency of left lower limb functional jerks had decreased by approximately 50%, although mild intermittent jerking persisted during periods of legal dispute stress. The patient demonstrated partial insights into the connection between psychological stress and limb jerking and remained engaged in physical therapy.

## 3. Discussion

This case illustrates the complex diagnostic landscape surrounding the coexistence of epilepsy and FDS in resource‐limited clinical environments. The primary diagnostic challenge resided in avoiding the trap of electroclinical dissociation assuming that because an EEG displays epileptiform activity during a clinical movement, the movement must be epileptic in origin.

### 3.1. Mechanistic Interpretation of Electroclinical Dissociation

Physiologically, a generalized 3–4 Hz spike‐and‐wave discharge cannot generate persistent, isolated unilateral limb jerking while leaving consciousness and contralateral limb control entirely intact. In our patient, the generalized discharges reflect his background GGE traits. The temporal overlap between these interictal/subclinical epileptiform bursts and the functional limb jerks represents a chance co‐occurrence. Recognizing electroclinical dissociation requires looking beyond the presence of spikes to evaluate physiological congruence [11].

### 3.2. Application of Pediatric ILAE Frameworks and “Rule‐In” Diagnosis

Historically, functional symptoms were diagnosed by systematically excluding structural or metabolic diseases [7]. Modern frameworks, including the ILAE pediatric recommendations [7] and general FND guidelines [3, 4], mandate a positive “rule‐in” strategy [3, 4, 7]. In our patient, key “rule‐in” clinical signs included the following:

• Semiologically variable motor activity influenced by attention and distraction.
• Localization of symptoms strictly to a previously traumatized body part (left lower limb).
• Preserved awareness during prominent rhythmic/clonic‐like motor activity.
• Lack of response to multiple high‐dose ASMs.

### 3.3. Traumatic Precipitants and Predictive Processing Models

The onset of FDS specifically in the left leg following a road traffic accident aligns closely with modern neurobiological models of FND. Trauma and local physical pain act as potent “perceptual anchors” or triggers [9, 12]. Under predictive processing models, bodily injury increases top‐down attentional focus toward the affected limb. In vulnerable individuals such as adolescents with chronic illness, borderline cognitive capacity, and heightened psychosocial stress, this heightened focus generates abnormal priors (“expectations of abnormal movement”), resulting in involuntarily generated functional motor outputs [9, 11].

### 3.4. Clinical Correlates and Somatic Symptoms

The patient’s co‐occurring dysphagia, chronic headache, and neck pain are notable. Rather than indicating multiorgan organic disease, multiple somatic complaints frequently serve as “red flags” for central sensitization and somatic symptom amplification secondary to affective distress (PHQ‐9: 14, GAD‐7: 13) [3, 10]. Addressing these somatic correlates within a holistic biopsychosocial framework is essential for comprehensive recovery.

### 3.5. Limitations and Strategic Need for Extended Follow‐Up

A major limitation of this report is the short follow‐up duration (2 months). In FDS management, a 2‐month observation window is insufficient to confirm long‐term diagnostic stability, complete psychological recovery, or sustained reduction in healthcare utilization [8, 10]. Long‐term follow‐up over 6–12 months is necessary to monitor the gradual weaning of superfluous ASMs, evaluate the resolution of legal litigation stress, and assess the long‐term efficacy of physical rehabilitation and CBT [7, 10] (Table 1).

**Table 1:** Timelines.

Timeframe	Clinical event
4 years prior	Onset of epilepsy with poor academic performance
3 months prior	Road traffic accident with left lower limb injury
Following RTA	Persistent unilateral left lower limb jerking begins
Subsequent months	Multiple admissions; no response to multiple ASMs
Recent weeks	Evaluation for dysphagia, headache, neck pain
9 days prior	Posterior fossa decompression for Chiari I malformation
Current admission	Video‐EEG confirmation of electroclinical dissociation

### 3.6. Core Tips

• Adopt a positive “rule‐in” diagnostic approach: Do not treat FDS as a diagnosis of exclusion. Look for positive physical signs such as distractibility, semiological variability, and symptom localization to prior trauma sites.
• Beware of noncausal EEG spikes: Epileptiform activity on EEG proves that the patient has an epileptic diathesis, but it does not prove that the index event is epileptic. Always verify electroclinical congruence.
• Target SSRIs appropriately: Prescribe SSRIs/SNRIs in FDS patients only when clear comorbid depression or anxiety disorders are present, rather than as a primary treatment for functional motor movements.
• Communicate transparently: Frame FDS as a real, brain‐network “software” problem to build therapeutic alliance, reduce stigma, and prevent doctor‐shopping or dangerous ASM escalation.

**Nomenclature**
ADLs: Activities of daily living
ASM: Antiseizure medication
EPC: Epilepsia partialis continua
FDS: Functional dissociative seizures
FND: Functional neurological disorder
GGE: Genetic generalized epilepsy
GSWDs: Generalized spike–wave discharges
GTCS: Generalized tonic‐clonic seizures
ILAE: International League Against Epilepsy
PNES: Psychogenic nonepileptic seizures
Video‐EEG: Video‐electroencephalography

## Author Contributions

Y.Y.A. conceived, wrote, and edited the manuscript.

## Funding

No financial support was received for this study.

## Disclosure

The author read and approved the final version.

## Ethics Statement

Ethical approval was not required for this single‐patient case report. The case was managed in accordance with the Declaration of Helsinki. Written informed consent for publication was obtained from the patient’s guardian.

## Consent

Written informed consent for publication of clinical details and accompanying images was obtained from the patient’s legal guardian.

## Conflicts of Interest

The author declares no conflicts of interest.

## Patient Perspective

“My family and I were confused because the leg jerks kept getting worse every time the seizure medications were increased. Understanding that the leg jerks were caused by the stress of the accident and lawsuit and were separate from my main epilepsy brought huge relief. Knowing my brain wasn’t damaged further helped me focus on my physical exercises.”

## Data Availability

The data that support the findings of this study are available from the corresponding author upon reasonable request.
